# Produce D-allulose from non-food biomass by integrating corn stalk hydrolysis with whole-cell catalysis

**DOI:** 10.3389/fbioe.2023.1156953

**Published:** 2023-02-24

**Authors:** Qing Jia, Hui Zhang, Anqi Zhao, Lingbo Qu, Wenlong Xiong, Md. Asraful Alam, Jixing Miao, Weigao Wang, Feihu Li, Jingliang Xu, Yongkun Lv

**Affiliations:** ^1^ School of Chemical Engineering, Zhengzhou University, Zhengzhou, China; ^2^ School of Life Sciences, Zhengzhou University, Zhengzhou, China; ^3^ Department of Chemical Engineering, Shriram Center, Stanford University, Stanford, CA, United States; ^4^ State Key Laboratory of Fine Chemicals, Dalian University of Technology, Dalian, China

**Keywords:** D-allulose, corn stalk, valorization, non-food feedstock, whole-cell catalyst

## Abstract

D-allulose is a high-value rare sugar with many health benefits. D-allulose market demand increased dramatically after approved as generally recognized as safe (GRAS). The current studies are predominantly focusing on producing D-allulose from either D-glucose or D-fructose, which may compete foods against human. The corn stalk (CS) is one of the main agricultural waste biomass in the worldwide. Bioconversion is one of the promising approach to CS valorization, which is of significance for both food safety and reducing carbon emission. In this study, we tried to explore a non-food based route by integrating CS hydrolysis with D-allulose production. Firstly we developed an efficient *Escherichia coli* whole-cell catalyst to produce D-allulose from D-glucose. Next we hydrolyzed CS and achieved D-allulose production from the CS hydrolysate. Finally we immobilized the whole-cell catalyst by designing a microfluidic device. Process optimization improved D-allulose titer by 8.61 times, reaching 8.78 g/L from CS hydrolysate. With this method, 1 kg CS was finally converted to 48.87 g D-allulose. This study validated the feasibility of valorizing corn stalk by converting it to D-allulose.

## 1 Introduction

The excessive intake of high energy sugars has caused many health problems, such as diabetes, hypertension, hyperlipidemia, and other diseases (Xia et al., 2021). D-allulose (D-psicose or D-ribo-2-hexulose) is a good sugar substitute, because it is an low calorie sweeter (70% sweetness of sucrose), and inert in energy metabolism (Iida et al., 2010). Besides, D-allulose also has many other health benefits, such as anti-oxidative, anti-diabetic (Hossain et al., 2015), anti-obesity (Nagata et al., 2015), and neuroprotective effects, and others (Juneja et al., 2019). The approval of D-allulose as GRAS by FDA has stimulated its market demand as food ingredient and dietary supplement (Zhang W. L. et al., 2017). However, as a rare sugar, D-allulose is hardly found in nature (Mu et al., 2011). Very small quantities of D-allulose can be found in the stems or leaves of Itea and wheat and some bacteria (Zhang et al., 2016b).

In the past decades, both chemical (Doner, 1979) and biological (Mu et al., 2011; Zhu et al., 2012) methods have been developed to synthesize D-allulose. Among them, biological methods are more attractive, because it is greener, lower cost, and its product is easier to purify (Izumori, 2002). The currently two most studied bioproduction approaches are the aldol condensation pathway and the Izumoring strategy (Zhang W. L. et al., 2017). In the aldol condensation pathway, D-allulose is produced by L-rhamnulose-1-phosphate aldolase-catalyzed condensation of dihydroxyacetone phosphate and D-glyceraldehyde and subsequent dephosphorylation (Li et al., 2017). This pathway involves the cofactor (ATP and NAD(P)^+^) regeneration and at least 5 functional genes (from glycerol to D-allulose) (Brovetto et al., 2011; Li et al., 2011; Wei et al., 2015). In comparison, the Izumoring strategy is simpler and straightforward. D-allulose production from D-glucose utilizes only 2 enzymes: D-glucose isomerase (interconversion of D-glucose and D-fructose) and D-psicose 3-epimerase (interconversion of D-fructose and D-allulose), and involves no cofactor regeneration (Izumori, 2006; Men et al., 2014). Consequently, the Izumoring strategy is the predominantly explored approach (Li et al., 2015; Zhang W. et al., 2017; Chen et al., 2017; Zhang et al., 2020). Based on this strategy, either D-fructose (Zhang et al., 2013; Zhang et al., 2015; Zhang et al., 2016a; He et al., 2016) or D-glucose (Men et al., 2014) was used as the substrate for D-allulose production.

With the continues increase of world population, food and resource insufficiency is becoming a great challenge to human society. The large scale commercial production of D-allulose from D-glucose or D-fructose (D-fructose is also produced from D-glucose) may “struggle” for food against human (Juneja et al., 2019). Consequently, developing non-food based and sustainable D-allulose producing process is necessary (Song et al., 2017). Corn stalk (CS) is a very promising alternative to the current D-glucose feedstock, because it is continuously produced as one of the major agricultural wastes and composed of 30%–40% cellulose, 20%–30% hemicellulose, and 10%–20% lignin (Yang et al., 2021). Cellulose is a polymer of glucose, and can be hydrolyzed into monomer (D-glucose). Among the annual lignocellulose output (about 170 billion tons), only 3% has been efficiently utilized (Liu et al., 2021; Shen and Sun, 2021). Compared with the free-enzyme reactions, whole-cell catalysis has the advantages of without tedious and costly enzyme purification process, protecting enzymes from harsh reaction conditions, enhancing reactions by colocalizing multiple enzymes within the cell, and preventing intermediates from diffusion (Chen et al., 2022). In this study, we will try to develop an efficient whole-cell catalyst to produce D-allulose from CS hydrolysate. By integration the whole-cell catalysis with CS hydrolysis, this study will provide a sustainable process for D-allulose production from the non-food biomass, as well as the valorization of agricultural waste CS.

## 2 Materials and methods

### 2.1 Genes, plasmids, and strains

The encoding genes of glucose isomerase from *Acidothermus cellulolyticus* 11B (AcceGI, NCBI access number: WP_011720899) (Mu et al., 2012) and D-psicose 3-epimerase (CcDPEase, NCBI access number: 3VNI_A) (Mu et al., 2011) were codon optimized and synthesized by Sangon Biotech (Shanghai, China). Other putative glucose isomerase, xylose isomerase, and D-psicose 3-epimerase genes were obtained by bioinformatic analysis and amplified from corresponding genome DNA (Table 1). The ePathBrick plasmid pET-28a (PB) was used for gene expression, fusion gene construction, and gene copy number optimization (Xu et al., 2012; Lv et al., 2017). *E. coli* JM109 was used for plasmid construction, maintenance, and propagation. *E. coli* BL21 (DE3) was used for protein expression, whole-cell catalyst development, and cell immobilization.

**TABLE 1:** Glucose isomerase, xylose isomerase, and D-psicose 3-epimerase genes discovered by bioinformatic analysis.

Gene	Enzyme	Original strain	Length (bp)
*YlXI*	Xylose isomerase	*Yarrowia lipolytica* Po1f	1,179
*YlGPI*	Glucose-6-phosphate isomerase	*Yarrowia lipolytica* Po1f	1,668
*GoDPEase*	D-psicose 3-epimerase or xylose isomerase^(^ ^a^ ^)^	*Gluconobacter oxydans* 621H	852
*GoXI_02*	Xylose isomerase	*Gluconobacter oxydans* 621H	744
*GoGPI*	Glucose-6-phosphate isomerase	*Gluconobacter oxydans* 621H	1,068
*PpDPEase_01*	D-psicose 3-epimerase	*Pseudomonas putida* KT2440	1,248
*PpDPEase_02*	D-psicose 3-epimerase	*Pseudomonas putida* KT2440	783
*PpXI*	Xylose isomerase	*Pseudomonas putida* KT2440	816
*BsDPEase_01*	D-psicose 3-epimerase	*Bacillus subtilis* subsp. subtilis str. 168	915
*BsDPEase_02*	D-psicose 3-epimerase	*Bacillus subtilis* subsp. subtilis str. 168	894
*BsXI*	Xylose isomerase	*Bacillus subtilis* subsp. subtilis str. 168	1,338
*BsGPI*	Glucose-6-phosphate isomerase	*Bacillus subtilis* subsp. subtilis str. 168	1,353
*PaXI*	Xylose isomerase	*Pseudomonas aeruginosa* PAO1	816
*PaDPEase_01*	D-psicose 3-epimerase	*Pseudomonas aeruginosa* PAO1	783
*PaDPEase_02*	D-psicose 3-epimerase	*Pseudomonas aeruginosa* PAO1	798
*BtDPEase*	D-psicose 3-epimerase or xylose isomerase^(^ ^a^ ^)^	*Bacillus thuringiensis* ATCC 10792	843

^a^ These genes were annotated as both D-psicose 3-epimerase and xylose isomerase in the local blast analysis.

### 2.2 Bioinformatic analysis

The genome sequences of *Yarrowia lipolytica* strain CLIB89(W29), *Bacillus subtilis* subsp. subtilis str. 168 (NC_000964.3), *Bacillus thuringiensis* strain ATCC 10792 (NZ_CP021061.1), *Gluconobacter oxydans* 621H (NC_006677.1), *Pseudomonas aeruginosa* PAO1 (NC_002516.2), and *Pseudomonas putida* KT2440 (NC_002947.4) were downloaded from NCBI. The amino acid sequences of all glucose isomerase, xylose isomerase, and D-psicose 3-epimerase were downloaded from NCBI (update 5 July 2020) and used to query the genome sequences using TBLASTN. The predicted genes were double checked by querying the amino acid sequences of the corresponding enzymes.

### 2.3 Molecular biology

*AcceGI* and *CcDPEase* were subcloned into pET-28a (PB) between *Bam*HI and *Hind*III sites to yield pET28a (PB)-AcceGI and pET28a (PB)-CcDPEase, respectively. The putative genes (Table 1) were subcloned into pET-28a (PB) by homologous one-step cloning, resulting in corresponding recombinant plasmids (Table 2) (Xu et al., 2012; Lv et al., 2017). The primers were flanked with homologous sequence of pET-28a (PB) at 5′-terminal (Supplementary Table S1). The homologous one-step cloning was carried out using ClonExpress II One Step Cloning Kit (Vazyme, Nanjing, China) (Zhang et al., 2014).

**TABLE 2:** Plasmids used in this study.

Plasmid	Genetic characteristics	Reference or source
pET-28a (PB)	An ePathBrick vector	Xu et al. (2012), Lv et al. (2017)
pET28a (PB)-AcceGI	pET-28a (PB) carrying a glucose isomerase gene *AcceGI* from *Acidothermus cellulolyticus* 11B	This study
pET28a (PB)-CcDPEase	pET-28a (PB) carrying a D-psicose 3-epimerase gene *CcDPEase* from *Clostridium cellulolyticum* H10	This study
pET28a (PB)-YlXI	pET-28a (PB) carrying a putative xylose isomerase gene from *Yarrowia lipolytica* Po1f	This study
pET28a (PB)-YlGPI	pET-28a (PB) carrying a putative glucose-6-phosphate isomerase gene from *Yarrowia lipolytica* Po1f	This study
pET28a (PB)-GoDPEase	pET-28a (PB) carrying a putative D-psicose 3-epimerase or xylose isomerase^(^ ^a^ ^)^ gene from *Gluconobacter oxydans* 621H	This study
pET28a (PB)-GoXI_02	pET-28a (PB) carrying a putative xylose isomerase gene from *Gluconobacter oxydans* 621H	This study
pET28a (PB)-GoGPI	pET-28a (PB) carrying a putative glucose-6-phosphate isomerase gene from *Gluconobacter oxydans* 621H	This study
pET28a (PB)-PpDPEase_01	pET-28a (PB) carrying a putative D-psicose 3-epimerase gene from *Pseudomonas putida* KT2440	This study
pET28a (PB)-PpDPEase_02	pET-28a (PB) carrying a putative D-psicose 3-epimerase gene from *Pseudomonas putida* KT2440	This study
pET28a (PB)-PpXI	pET-28a (PB) carrying a putative xylose isomerase gene from *Pseudomonas putida* KT2440	This study
pET28a (PB)-BsDPEase_01	pET-28a (PB) carrying a putative D-psicose 3-epimerase gene from *Bacillus subtilis* subsp. *subtilis* str. 168	This study
pET28a (PB)-BsDPEase_02	pET-28a (PB) carrying a putative D-psicose 3-epimerase gene from *Bacillus subtilis* subsp. *subtilis* str. 168	This study
pET28a (PB)-BsXI	pET-28a (PB) carrying a putative xylose isomerase gene from *Bacillus subtilis* subsp. *subtilis* str. 168	This study
pET28a (PB)-BsGPI	pET-28a (PB) carrying a putative glucose-6-phosphate isomerase gene from *Bacillus subtilis* subsp. *subtilis* str. 168	This study
pET28a (PB)-PaXI	pET-28a (PB) carrying a putative xylose isomerase gene from *Pseudomonas aeruginosa* PAO1	This study
pET28a (PB)-PaDPEase_01	pET-28a (PB) carrying a putative D-psicose 3-epimerase gene from *Pseudomonas aeruginosa* PAO1	This study
pET28a (PB)-PaDPEase_02	pET-28a (PB) carrying a putative D-psicose 3-epimerase gene from *Pseudomonas aeruginosa* PAO1	This study
pET28a (PB)-BtDPEase	pET-28a (PB) carrying a putative D-psicose 3-epimerase or xylose isomerase^(^ ^a^ ^)^ gene from *Bacillus thuringiensis* ATCC 10792	This study
pET28a (PB)-GP	pET-28a (PB) carrying *AcceGI* and *CcDPEase* in fusion form	This study
pET28a (PB)-GSP	pET-28a (PB) carrying *AcceGI* and *CcDPEase* linked with “GGGGS” encoding sequence	This study
pET28a (PB)-GS_2_P	pET-28a (PB) carrying *AcceGI* and *CcDPEase* linked with “GGGGSGGGGS” encoding sequence	This study
pET28a (PB)-GS_3_P	pET-28a (PB) carrying *AcceGI* and *CcDPEase* linked with “GGGGSGGGGSGGGGS” encoding sequence	This study
pET28a (PB)-GEP	pET-28a (PB) carrying *AcceGI* and *CcDPEase* linked with “EAAAK” encoding sequence	This study
pET28a (PB)-GE_2_P	pET-28a (PB) carrying *AcceGI* and *CcDPEase* linked with “EAAAKEAAAK” encoding sequence	This study
pET28a (PB)-GE_3_P	pET-28a (PB) carrying *AcceGI* and *CcDPEase* linked with “EAAAKEAAAKEAAAK” encoding sequence	This study
pET28a (PB)-AcceGI-CcDPEase	pET-28a (PB) carrying *AcceGI* and *CcDPEase* in monocistronic form	This study
pET28a (PB)-AcceGI_×2_-CcDPEase	pET-28a (PB) carrying 2-copy *AcceGI* and *CcDPEase* in monocistronic form	This study
pET28a (PB)-AcceGI-CcDPEase_×2_	pET-28a (PB) carrying *AcceGI* and 2-copy *CcDPEase* in monocistronic form	This study
pET28a (PB)-AcceGI_×3_-CcDPEase	pET-28a (PB) carrying 3-copy *AcceGI* and *CcDPEase* in monocistronic form	This study
pET28a (PB)-AcceGI_×4_-CcDPEase	pET-28a (PB) carrying 4-copy *AcceGI* and *CcDPEase* in monocistronic form	This study
pET28a (PB)-AcceGI_×5_-CcDPEase	pET-28a (PB) carrying 5-copy *AcceGI* and *CcDPEase* in monocistronic form	This study
pET28a (PB)-AcceGI_×6_-CcDPEase	pET-28a (PB) carrying 6-copy *AcceGI* and *CcDPEase* in monocistronic form	This study
pET28a (PB)-AcceGI_×7_-CcDPEase	pET-28a (PB) carrying 7-copy *AcceGI* and *CcDPEase* in monocistronic form	This study

^a^ These genes were annotated as both D-psicose 3-epimerase and xylose isomerase in the local blast analysis.

AcceGI and CcDPEase were fused together directedly or linked with flexible or rigid linkers. The detailed processes were as follows. AcceGI_GP was amplified with primer pair GI_GP_Fusion F/GI_GP_Fusion R, and subcloned into pET28a (PB)-CcDPEase at *Bam*HI site. The resulting plasmid pET28a (PB)-GP will produce a fusion protein GP, which links the C-terminal of AcceGI to the N-terminal of CcDPEase directly. AcceGI_GS1P was amplified with primer pairs GI_GP_Fusion F/GI_GS1P_Fusion R, and subcloned into pET28a (PB)-CcDPEase at *Bam*HI site. The resulting plasmid pET28a (PB)-GS1P will produce a fusion protein GS1P, which links AcceGI and CcDPEase with linker GGGGS. AcceGI_GS2P and CcDPEase_GS2P were amplified with primer pairs GI_GP_Fusion F/GS2P R1 and GS2P F2/DPEase_GP_Fusion R2, and subcloned into pET-28a (PB) between *Bam*HI and *Hind*III sites. The resulting plasmid pET28a (PB)-GS2P will produce a fusion proteins GS2P, which links AcceGI and CcDPEase with linkers GGGGSGGGGS. AcceGI_GS3P and CcDPEase_GS3P were amplified with primer pairs GI_GP_Fusion F/GS3P R1 and GS3P F2/DPEase_GP_Fusion R2, and subcloned into pET-28a (PB) between *Bam*HI and *Hind*III sites. The resulting plasmid pET28a (PB)-GS3P will produce a fusion proteins GS3P, which links AcceGI and CcDPEase with linkers GGGGSGGGGSGGGGS. Plasmids pET28a (PB)-GE1P, pET28a (PB)-GE2P, and pET28a (PB)-GE3P were constructed following the same process, except that AcceGI_GE1P, AcceGI_GE2P, CcDPEase_GE2P, AcceGI_GE3P, and CcDPEase_GE3P, were amplified with primer pairs GI_GP_Fusion F/GI_GE1P_Fusion R, GI_GP_Fusion F/GE2P R1, GE2P F2/DPEase_GP_Fusion R2, GI_GP_Fusion F/GE3P R1, and GE3P F2/DPEase_GP_Fusion R2 (Supplementary Table S1; Table 2). All the above subcloning were carried out using ClonExpress II One Step Cloning Kit (Vazyme, Nanjing, China). All the encoding genes of flexible linker ((GGGGS)_n_ (n = 1–3)) and rigid linker ((EAAAK)_n_ (n = 1–3)) were codon optimized with GenSmart™ Codon Optimization online tool (https://www.genscript.com/gensmart-free-gene-codon-optimization .html) (Supplementary Table S2) (Guo et al., 2013).

To co-overexpress glucose isomerase and D-psicose epimerase in single cell, AcceGI and CcDPEase were assembled into monocistronic form by using isocaudomers (*Avr*II, *Nhe*I, and *Sal*I) and subsequent T4 ligation (Xu et al., 2012). The gene copy number optimization was also carried out by using this method.

### 2.4 Protein expression and enzymatic activity analysis

The recombinant protein expression was carried out following previous methods with moderate modification (Lv et al., 2017). A *E. coli* BL21 (DE3) colony containing corresponding recombinant plasmid was inoculated into a sterile incubating tube containing 2 mL LB medium. After overnight incubation at 37°C and 200 rpm, 1 mL culture was inoculated into 250 mL shaking flask containing 25 mL LB medium. The strain was cultured at 37°C and 200 rpm until OD_600_ reach 0.6–0.8. Cool down the culture to 25°C, and add 0.5 mM isopropyl-β-D-thiogalactopyranoside (IPTG) for induction. After another 8 h incubation at 25°C and 200 rpm, centrifuge at 4°C and 8,000 rpm to collect cell pellet. After wash with pure water, the cells were resuspended in water, and used for SDS-PAGE assay, enzymatic activity analysis, process optimization, or immobilization.

The biomass was measured by recording the optical density at 600 nm (OD_600_) with a Tecan Infinite^®^ M Plex microplate reader (Tecan, Männedorf, Switzerland). SDS-PAGE (Sangon Biotech, Shanghai, China) was used to validate the recombinant protein expression. The enzymatic activities of AcceGI and CcDPEase were measured following previous methods (Mu et al., 2011; Mu et al., 2012).

### 2.5 Analytical methods

D-glucose, D-fructose, and D-allulose were analyzed with a Thermo Scientific™ Dionex™ ICS-6000 ion chromatography system (HPIC) equipped with a Dionex CarboPac™ PA20 BioLC™ 3 × 150 mm analytical column. The mobile phase A was pure water, and B was water with 200 mM NaOH. The gradient (B%) was as follows: 0–15 min 10%, 15.1–25 min 100%, 25.1–35 min 10%. The flow rate was 0.5 mL/min. The oven and detector temperature was maintained at 30°C. The inject volume was 25 μL.

### 2.6 *Escherichia coli* cell immobilization

The recombinant cells were cultured and collected as the above description, and mixed with sodium alginate solutions of different concentrations (0.1%, 0.5%, 1.0%, 1.5%, 2.0%, w/v). The mixture was then pumped into calcium chloride solutions of different concentrations (1.0%, 1.5%, 2.0%, 2.5%, 3.0%, w/v). The flow rate was 60 mL/h. Incubate at room temperature for different period (20 min, 40 min, 60 min, 80 min, 100 min) to obtain calcium alginate fibers containing recombinant cells.

### 2.7 Corn stalk pretreatment

The CS was collected from local farm (Yuanyang county, Henan province, China, 113.9^o^ *E*, 35.1^o^ *N*), and then totally dried, ground and screened with a 60 mesh sieve. The pulping was carried out by cooking with 2% (w/v) NaOH solution at 80°C and atmospheric pressure for 2 h. The ratio of CS to NaOH was 1:20 (w/w). Wash the pulp with pure water until pH maintain stable, then totally dry the pulp in oven at 105°C. The hydrolysis was carried out with cellulases (Qingdao Vland Biotech Inc., Qingdao, China) in deionized water (adjust to pH5.0 with acetic acid). It should be noted that the hydrolysis buffer should not contain any sodium ion, which will cause the calcium alginate fiber instable in the next step. A final concentration of 10% (w/v) substrate and (10 FPU cellulase)/(g substrate) was added. Incubate the mixture at 50°C and 200 rpm for 3 days for sufficient hydrolysis.

### 2.8 Process optimization

The process optimization was carried out by single factor optimization. Recombinant protein expression, induction time (4–20 h) and IPTG concentration (0.001–0.5 mM) were optimized stepwise following the previous descriptions (Lv et al., 2017). For cell immobilization, calcium chloride concentration (1.0%–3.0%), sodium alginate concentration (0.1%–2.0%), cell dosage (OD_600_ = 10–35), and immobilization time (20–100 min) were optimized stepwise. For the D-allulose production, D-glucose concentration (20–60 g/L), reaction temperature (55–75°C), and reaction time (2–12 h) were optimized stepwise. All the bioconversions were carried out in pure water at natural pH (around 7.5). For each bioconversion, triplicated biological repeats were carried out in 250-mL shaking flasks. The statical analysis and graphing were performed using Origin Lab software (OriginLab Corporation, Northampton, MA).

## 3 Results

### 3.1 Producing D-allulose from glucose by developing whole-cell catalysts

**FIGURE 1:**
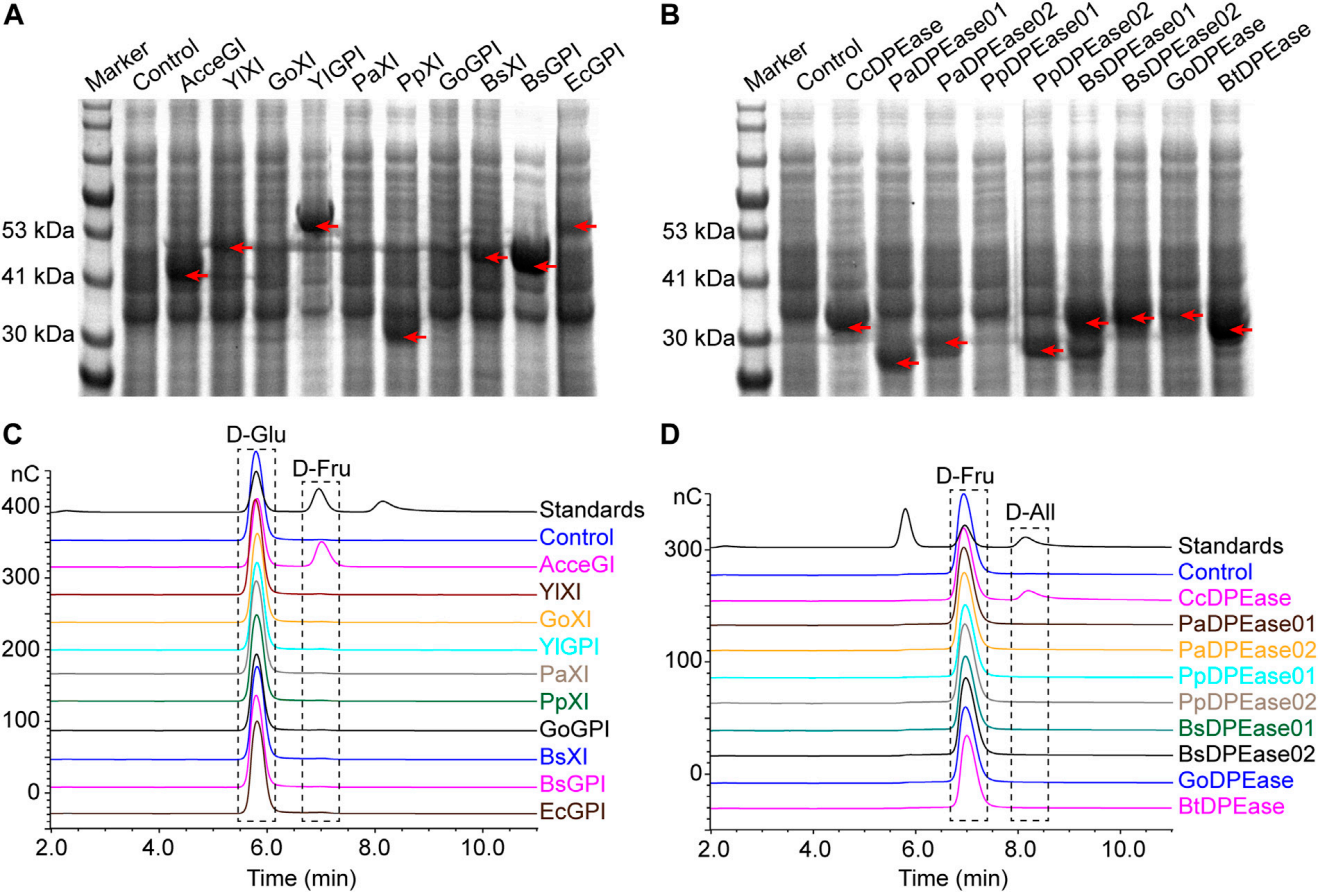
Expression and activity analysis of predicted glucose isomerase, xylose isomerase, and D-psicose 3-epimerase. **(A)** Expression of predicted glucose isomerase and glucose isomerase **(B)** Expression of predicted D-psicose 3-epimerase. **(C)** Enzyme activity analysis of predicted glucose isomerase and glucose isomerase. The analysis was carried out by converting D-glucose to D-fructose **(D)** Enzyme activity analysis of predicted D-psicose 3-epimerase. The analysis was carried out by converting D-fructose to D-allulose. In all panels, control refers to *E. coli* BL21 (DE3) containing empty pET-28a (PB) plasmid. In SDS-PAGE results, red arrows indicate the recombinant proteins. In HPIC results, standards refer to mixture of D-glucose, D-fructose, and D-allulose standards.

We firstly explored the potential glucose isomerase and D-psicose 3-epimerase in *Yarrowia lipolytica* strain CLIB89(W29), *Bacillus subtilis* subsp. subtilis str. 168, *Bacillus thuringiensis* strain ATCC 10792, *Gluconobacter oxydans* 621H, *Pseudomonas aeruginosa* PAO1, and *Pseudomonas putida* KT2440 by using local TBLASTN method (Altschul et al., 1997). We chose these microbes because they are readily available in our laboratory. Previous studies showed that many xylose isomerases also show glucose isomerase activities, consequently we also analyzed the xylose isomerases in these microbes (Mu et al., 2012; Karaoglu et al., 2013). The bioinformatic analysis predicted 3 glucose-6-phosphate isomerases, 7 xylose isomerases, and 8 D-psicose 3-epimerases, among which 2 were annotated as both D-psicose 3-epimerase and xylose isomerase (Table 1). To validate the activities of these putative genes, we overexpressed them in *E. coli* BL21 (DE3). Besides, the known glucose isomerase from *Acidothermus cellulolyticus* 11B (AcceGI) and D-psicose 3-epimerase from *Clostridium cellulolyticum* H10 (CcDPEase) were also overexpressed (Mu et al., 2011; Mu et al., 2012). SDS-PAGE results clearly showed that almost all these genes were successfully expressed, except GoXI, PaXI, GoGPI, and PpDPEase01 (Figures 1A, B). Unfortunately, in the subsequent enzymatic analysis, only AcceGI and CcDPEase showed obvious glucose isomerase and D-psicose 3-epimerase activities, respectively (Figures 1C, D).

**FIGURE 2:**
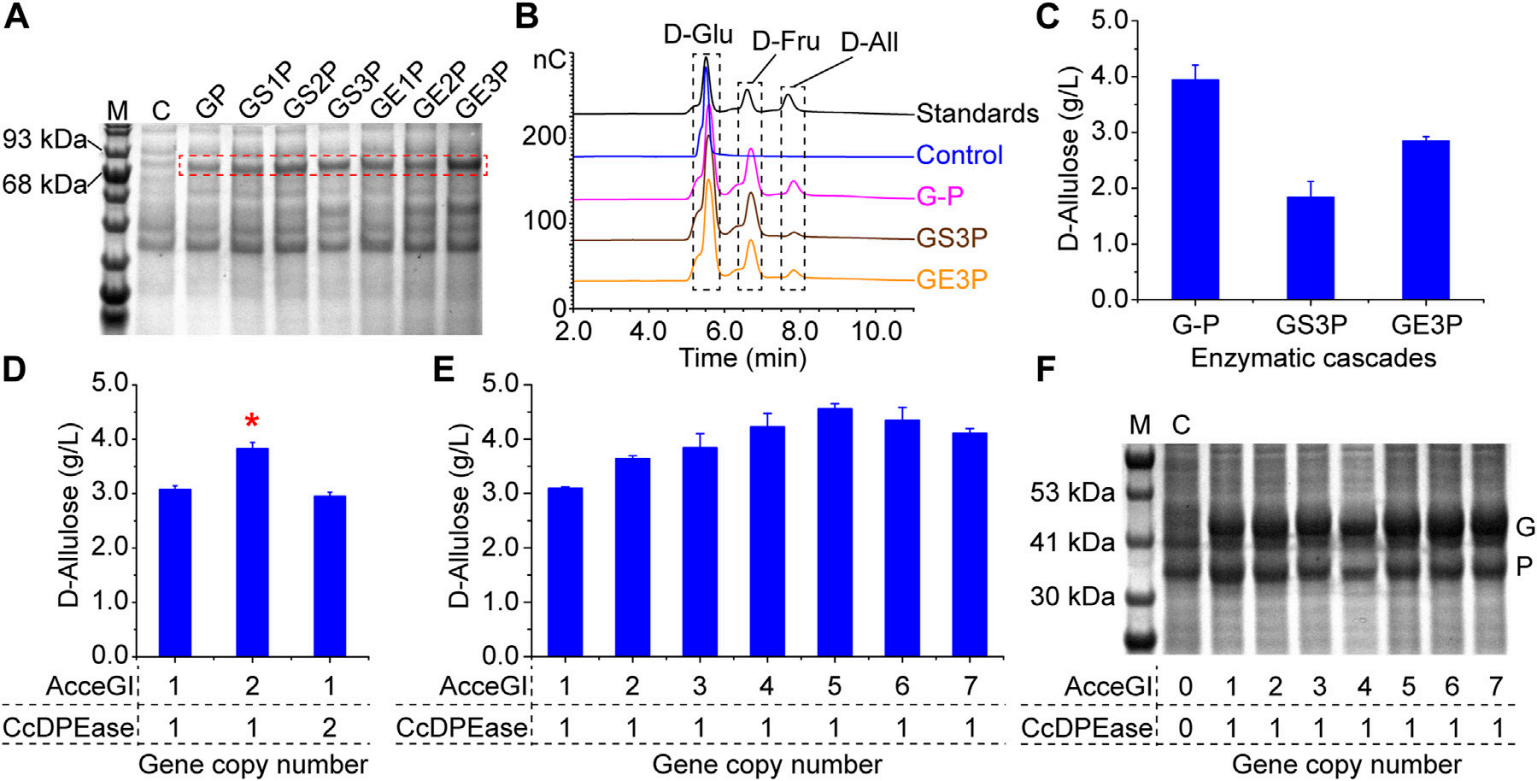
Optimization of the whole-cell catalyst. **(A)** Expression of the fusion enzymes. The calculated molecular weights were as follows: GP 81.49 kDa, GS1P 81.80 kDa, GS2P 82.11 kDa, GS3P 82.42 kDa, GE1P 81.96 kDa, GE2P 82.42 kDa, GE3P 82.89 kDa. M refers to marker. C refers to the control, which contain the empty pET-28a (PB) plasmid. **(B)** HPIC analysis of the enzymatic cascades **(C)** Efficiency of the enzymatic cascades in converting D-glucose to D-allulose. GP refers to the fusion protein linked AcceGI and CcDPEase directly. Other fusion protein are those linked AcceGI and CcDPEase through corresponding flexible or rigid linkers. G-P refers to the cascade composed of free AcceGI and CcDPEase. Standards refer to the D-glucose, D-fructose, and D-allulose mixture. **(D)** Analysis of rate limiting step of the enzymatic cascade by stepwise increasing gene copy number **(E)** Improving the activity of the whole-cell catalyst by optimizing the enzyme expression levels. **(F)** Expression level analysis of AcceGI and CcDPEase. M refers to marker. C refers to the control, which contain the empty pET-28a (PB) plasmid. G indicates the bands of AcceGI; P indicates the bands of CcDPEase.

To develop the one-step D-allulose producing whole-cell catalyst, we co-overexpressed AcceGI and CcDPEase in monocistronic form (G-P) in 1 cell by using the ePathBrick method (Xu et al., 2012). When incubating the whole-cell catalyst (G-P) with 50 g/L D-glucose, 3.94 g/L D-allulose was produced with a yield of 7.88% (Figures 2B, C).

### 3.2 Improving the activity of whole-cell catalyst by balancing enzyme expression level

Spatially confining enzymes is a commonly used approach to preventing intermediates diffusion and thus improving enzymatic cascade efficiency (Zhong et al., 2022). Consequently, we designed a panel of fusion proteins, which linked AcceGI and CcDPEase directly or through flexible or rigid linkers. The expression of fusion proteins was validated by SDS-PAGE analysis (Figure 2A). The enzymatic activity analysis showed that all these fusion enzymes maintained their native activities. Moreover, longer linkers conferred enzymes with higher activities, which should be a result of less steric hindrance (Supplementary Figure S1). Consequently, we used the two best performing fusion proteins (GS3P and GE3P) to validate the enzymatic cascade. HPIC results showed that the fusion enzymes (GS3P and GE3P) directly converted D-glucose to D-allulose (Figure 2B). However, neither of the fusion enzymes performed better than that of the free enzyme cascade (Figure 2C). We deem this to be resulted from the steric hindrance effect, because the enzymes with longer linkers showed higher activities and the rigid linker performed better than the flexible one (Supplementary Figure S1) (Chen et al., 2013). These results indicated that the intermediate diffusion should not to be the predominant rate limiting factor in this whole-cell catalyst.

We next tried to improve the activity of the whole-cell catalyst by balancing the expression levels of the enzymes. This was achieved by optimizing gene copy numbers by using the ePathBrick method (Xu et al., 2012). The results showed that increasing AcceGI copy number improved the whole-cell activity by 24.38%, while increasing CcDPEase copy number had no effect on the whole-cell catalyst (Figure 2D). This result indicated that AcceGI is the rate limiting step of the enzymatic cascade. Subsequently we used this straightforward method to further stepwise increase the gene copy number to 7. The results showed that the activities of the while-cell catalyst improved along with the increasing of the AcceGI copy number until 5, and thereafter activities decreased (Figure 2E). The improvement of AcceGI expression level was validated with SDS-PAGE analysis (Figure 2F). Moreover, improving AcceGI copy number did not have obvious affect on the biomass (Supplementary Figure S2). Consequently, we used the optimal whole-cell catalyst containing 5 AcceGI (AcceGI_×5_-CcDPEase) in the subsequent research.

### 3.3 Producing D-allulose from corn stalk

**FIGURE 3:**
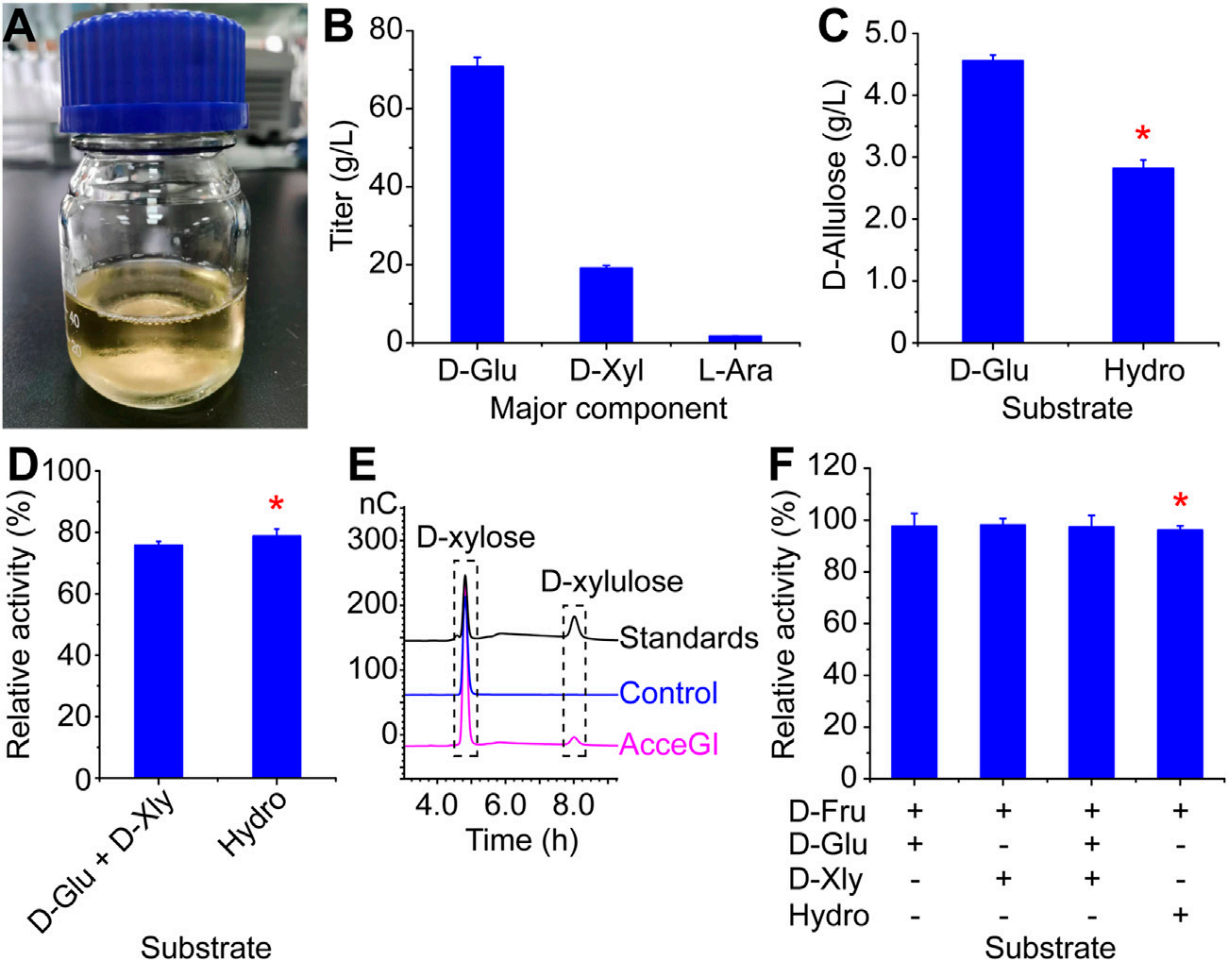
Producing D-allulose from CS. **(A)** CS hydrolysate by subsequential grind, alkaline pretreatment, and digestion **(B)** Major monosaccharide components of the CS hydrolysate. **(C)** D-allulose titers from pure D-glucose and CS hydrolysate **(D)** Relative activities of free AcceGI to D-glucose and D-xylose mixture and CS hydrolysate. The activity of free AcceGI to pure D-glucose was defined as 100%. **(E)** D-xylose can be used as the substrate of AcceGI, and converted to D-xylulose **(F)** Relative activities of free CcDPEase to mixtures of monosaccharides. The activity of free CcDPEase to pure D-fructose was defined as 100%. D-Glu refers to D-glucose. D-Xyl refers to D-xylose. L-Ara refers to L-arabinose. Hydro refers to CS hydrolysate. When using CS hydrolysate as substrate, the performance conditions were identical to those using D-glucose as substrate. The detailed performance conditions were described in the materials and methods section. Red stars indicate the effects of CS hydrolysate to enzymes.

The purpose of this study is to produce D-allulose from CS. So we next developed a process to convert CS into monosaccharide by subsequential grind, alkaline pretreatment, and digestion. The resulting hydrolysate was a light yellow solution, containing 70.82 g/L D-glucose, 19.13 g/L D-xylose, 1.69 g/L L-arabinose, and other minor components (D-mannose, D-galactose, lignans, and furfural) (Figures 3A, B). The D-glucose yield from dry CS was 27.83%.

When using the best performing whole-cell catalyst (AcceGI_×5_-CcDPEase) to convert this hydrolysate, 2.82 g/L D-allulose was produced from 50 g/L D-glucose (from CS hydrolysate) with a yield of 5.64% (Figure 3C). This yield equals to 61.82% of that from pure D-glucose (Figure 3C). We deem the loss of catalytic activity to two reasons, the inhibition effect of inhibitory factors (such as furfural) and the competition effect of other potential substrates. For instance, the glucose isomerase has been shown being capable of utilizing both D-glucose and D-xylose (Patra and Bera, 2014). To validate our hypothesis, we mimicked the hydrolysate by adding D-xylose to D-glucose to the same final concentrations. The activities of free AcceGI and CcDPEase to pure D-glucose and D-fructose were defined as 100%, respectively. The results showed that adding D-xylose decreased AcceGI activity by 24.14%, which is similar to that of the CS hydrolysate (Figure 3D). Moreover, the HPIC results showed that free AcceGI converted D-xylose into D-xylulose, which indicated the competitive effect between D-xylose and D-glucose to AcceGI (Figure 3E). On the other hand, potential substrates did not have effect on CcDPEase activity (Figure 3F). These results indicated that the activity decrease when using CS hydrolysate as substate is predominantly resulted from competition between D-glucose and D-xylose.

### 3.4 Enhancing D-allulose production by developing and optimizing a recyclable catalytic fiber

**FIGURE 4:**
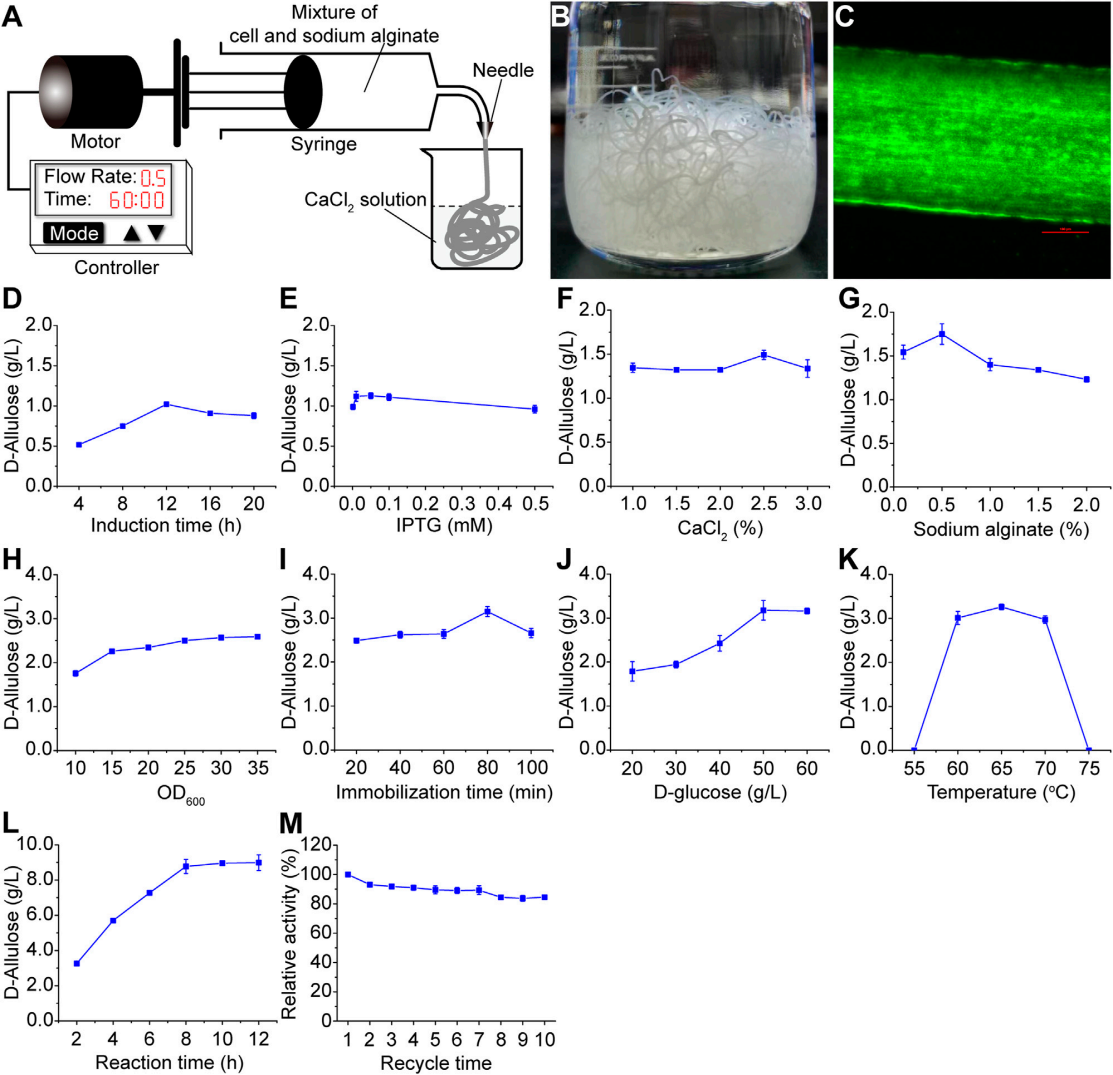
Whole-cell catalyst immobilization and optimization. **(A)** Architecture of the microfluidic systems **(B)** Calcium alginate fiber made by the microfluidic system. **(C)** Calcium alginate fiber containing fluorescent cells. This photo was taken with a fluorescent microscope. The cells were tracked by overexpressing EGFP. The red bar indicates 100 μm **(D)** Induction time and **(E)** IPTG concentration optimization for protein expression. **(F)** CaCl_2_ concentration, **(G)** sodium alginate concentration, **(H)** cell dosage, and **(I)** immobilization time optimization for the immobilization process. **(J)** Substrate concentration, **(K)** reaction temperature, and **(L)** reaction time optimization for the D-allulose production process **(M)** Reusability analysis of the calcium alginate fiber in D-allulose production. The detailed performance conditions were described in the materials and methods section.

To improve the whole-cell catalyst reusability and process economical efficiency, we designed a simple microfluidic system to immobilize the whole-cell catalyst in calcium alginate fiber. As shown in Figure 4A, a mixture of whole-cell catalyst and sodium alginate solution was in the syringe, which was driven by a motor. Calcium chloride solution in the beaker reacts with the fiber from the syringe, resulting in stable calcium alginate fibers (Figure 4B). The fiber’s diameter was controlled by the needle, and the flow rate was controlled by the controller. To validate the cell immobilization in the fiber, we substituted the enzymes (AcceGI and CcDPEase) with an enhanced green fluorescent protein (EGFP) to track the recombinant cells. Fluorescent microscope results showed that these cells were immobilized in the fibers, whose diameter was 500 μm (Figure 4C).

We next tried to improve the fiber’s activity by process optimization. The results showed that for the recombinant protein induction, the optimal induction time was 12 h, and optimal IPTG concentration was 0.05 mM (Figures 4D, E). For the immobilization process, the optimal concentrations of calcium chloride and sodium alginate were 2.5% (w/w) and 0.5% (w/w) respectively (Figures 4F, G), and the optimal immobilization time was 80 min (Figure 4I). Although higher D-allulose production was obtained along with higher cell dosage (OD_600_ = 10–35), however it increased very slow after OD_600_ reaching 15 (Figure 4H). As a result, we used OD_600_ = 15 in the subsequent optimization. For the D-allulose production process, the optimal substrate (D-glucose in CS hydrolysate) concentration and temperature were 50 g/L and 65°C (Figures 4J, K) respectively. Although higher D-allulose production was obtained along with longer reaction time during 2–12 h, however it increased very slow after 8 h (Figure 4L). As a result, we used 8 h as the reaction time in the subsequent reactions. Taking together, the process optimization improved D-allulose production by 8.61 times from 1.02 g/L to 8.78 g/L. To validate the fiber’s reusability, we reused one single fiber in 10 catalytic reactions. The results showed that the fiber maintained 84.53% relative activity after 10-cycle reuse (Figure 4M). It should be noted that calcium alginate fiber is unstable in the presence of some ions like sodium. Consequently, we did not optimize the reaction pH (avoiding the involvement of sodium buffer) and carried out all the bioconversions in pure water at natural pH (around 7.5). The results showed that the calcium alginate fiber was stable in pure water (Figure 4M). This not only stabilized the fiber, but also could be helpful for reducing the cost of future large scale applications (eliminating the involvement of any buffer).

## 4 Discussion

D-allulose is a GRAS sugar substitute, and characterized with many health benefits (Iida et al., 2010). As a rare sugar, D-allulose is hardly found in nature. The currently predominant production method is based on the Izumoring strategy, which use either D-glucose or D-fructose as substrate (Izumori, 2006; Juneja et al., 2019; Zhang et al., 2020). With the increasing concerns about insufficient food supply, exploring non-food based D-allulose producing method is becoming necessary (Hobbs, 2020). CS is one of the main agricultural wastes continuously produced worldwide. Dry CS contains more than 30% cellulose, which is a polymer of D-glucose (Zhang et al., 2021). Consequently, CS biomass could be a promising alternative feedstock to D-glucose or D-fructose.

The Izumoring strategy is limited by thermodynamic equilibrium. When the reaction reach equilibrium in the D-allulose production from D-glucose, the D-allulose yield was 18.18% (the ratio of D-glucose:D-fructose:D-allulose was 6.5:7:3) (Zhang W. et al., 2017). In the present study, 8.78 g/L D-allulose was produced from 50 g/L D-glucose (obtained by CS hydrolysis). The D-glucose yield from dry CS was 27.83%. Taking together, 1 kg CS was finally converted to 48.87 g D-allulose. This yield seemed very low. However, the D-allulose yield from D-glucose was 17.56% (8.78 g/L D-allulose from 50 g/L D-glucose in CS hydrolysate), which is close to the equilibrium state (18.18%).

The dry CS is mainly composed of cellulose, hemicellulose, and lignin. In this study, the hydrolysis yield three main monosaccharides (70.82 g/L D-glucose, 19.13 g/L D-xylose, and 1.69 g/L L-arabinose). Both D-glucose and D-xylose can be utilized by D-glucose isomerase, and the products are D-fructose and D-xylulose respectively (Mu et al., 2012). The substrate competition between D-xylose and D-glucose decreased D-fructose production by 24.14% (Figure 3D). This issue can be resolved by removing D-xylose from the solution or improving D-glucose isomerase’s substrate specificity. However, neither of them is easy to achieve. In the following research, we are going to simultaneously produce D-allulose and D-ribose, which is a important pentose with many valuable physiological functions (Mahoney et al., 2018; Li et al., 2021). D-ribose is currently produced from D-glucose through the pentose phosphate pathway (Cheng et al., 2017; Park et al., 2017). However, using this method 1 carbon is lost by releasing 1 CO_2_ for each D-ribose production, which is neither economical efficient or environmental friendly (Srivastava et al., 2012). By converting D-xylose to D-ribose (both of them are pentose) will not only explore novel D-ribose producing method, but also further improve the full utilization of CS.

## 5 Conclusion

In the present study, we produced D-allulose from CS by integrating the CS hydrolysis with a D-allulose producing whole-cell catalyst. By gene screening, co-overexpressing, copy number optimization, we obtained a high-efficient whole-cell catalyst, and used it to produce D-allulose from CS hydrolysate. We next designed a microfluidic system and used it to immobilize the whole-cell catalyst. After process optimization, the D-allulose titer improved by 8.61 times, reaching 8.78 g/L from CS hydrolysate. In this process, 1 kg CS was finally converted to 48.87 g D-allulose. The bioconversions were carried out in pure water. This study validated the feasibility of producing D-allulose from CS, a non-food feedstock.

## Data Availability

The original contributions presented in the study are included in the article/Supplementary Material, further inquiries can be directed to the corresponding authors.
