# Supplementary material for: Produce D-allulose from non-food biomass by integrating corn stalk hydrolysis with whole-cell catalysis
Source: Front Bioeng Biotechnol. 2023 Feb 24;11:1156953. doi: 10.3389/fbioe.2023.1156953 (PMC9998921; doi:10.3389/fbioe.2023.1156953)
Supplement: Supplementary file 1 [file DataSheet1.docx]

Supplementary materials

Produce D-allulose from non-food biomass by integrating corn stalk hydrolysis with whole-cell catalysis

Qing jia^1^, Hui Zhang^2^, Anqi Zhao^2^, Lingbo Qu^1^, Wenlong Xiong^1^, Md. Asraful Alam^1^, Jixing Miao^1^, Weigao Wang^3^, Feihu Li^1, 4^*, Jingliang Xu^1^*, and Yongkun Lv^1^*

1. School of Chemical Engineering, Zhengzhou University, No.100 Science Avenue, Zhengzhou, 450001, R. P. China;

2. School of Life Sciences, Zhengzhou University, No.100 Science Avenue, Zhengzhou, 450001, R. P. China;

3. Department of Chemical Engineering, Stanford University, 443 Via Ortega, Shriram Center, Palo Alto, California, 94305, United States;

4. State Key Laboratory of Fine Chemicals, Dalian University of Technology, Dalian 116024, P. R. China.

* Corresponding Authors:

Yongkun Lv (yklv@zzu.edu.cn)

Jingliang Xu (xujl@zzu.edu.cn)

Feihu Li (lifeihu@zzu.edu.cn)

**Table S1 Primers used in this study**

| **Primer** | **Sequence (5’ – 3’)** |
| --- | --- |
| YlXI F | TGGACAGCAAATGGGTCGCatgctcagactggcaaaccaaagc |
| YlXI R | tgctcgagtgcggccgcTTACCCGAAAAGAGCCTTGAAAGCG |
| YlGPI F | TGGACAGCAAATGGGTCGCatggctcagtccttcacgacc |
| YlGPI R | tgctcgagtgcggccgcTCAAGCGGCCCAAGCCTTGTACTGG |
| GoDPEase F | TGGACAGCAAATGGGTCGCatgacgaaactccccttccc |
| GoDPEase R | tgctcgagtgcggccgcTCAGGCCTCGCCGGTCCAGGCC |
| GoXI_02 F | TGGACAGCAAATGGGTCGCatgcagcatttcatgaagcgg |
| GoXI_02 R | tgctcgagtgcggccgcTCAACCGCACCGTTCTCGACTTCC |
| GoGPI F | TGGACAGCAAATGGGTCGCatgcccttcccggcgctgcaacc |
| GoGPI R | tgctcgagtgcggccgcTCACAAGCGTACGGCCGCACGTG |
| PpDPEase_01 F | TGGACAGCAAATGGGTCGCatgggcctcgaagaagttgtggcc |
| PpDPEase_01 R | tgctcgagtgcggccgcCTAGCAATTCGCTAAAACGGG |
| PpDPEase_02 F | TGGACAGCAAATGGGTCGCatgcctcgcttcgctgccaacc |
| PpDPEase_02 R | tgctcgagtgcggccgcTCAGATTGCGTTGTGGGTTTTCAGC |
| PpXI F | TGGACAGCAAATGGGTCGCatgactgatcgaattttttccc |
| PpXI R | tgctcgagtgcggccgcTCAGACCGCGGCCAATACCGCC |
| BsDPEase_01 F | TGGACAGCAAATGGGTCGCatggagttcggcgggatatggcg |
| BsDPEase_01 R | tgctcgagtgcggccgcTTACATGCTGAAGTATTTTGATACG |
| BsDPEase_02 F | TGGACAGCAAATGGGTCGCatgggcaaaaatgaaatcctgtgg |
| BsDPEase_02 R | tgctcgagtgcggccgcTTAAGCCAGATCCAACAACTGC |
| BsXI F | TGGACAGCAAATGGGTCGCatggctcaatctcattccagttc |
| BsXI R | tgctcgagtgcggccgcTTATACTTCTAAAATGTATTGG |
| BsGPI F | TGGACAGCAAATGGGTCGCatgacgcatgtacgctttgactac |
| BsGPI R | tgctcgagtgcggccgcTTAATCTTCCAGACGTTTTTCAAGC |
| PaXI F | TGGACAGCAAATGGGTCGCatggaatctaggattctttccc |
| PaXI R | tgctcgagtgcggccgcCTACAGGCGGGCGAGCAGCGCCC |
| PaDPEase_01 F | TGGACAGCAAATGGGTCGCatgccccgtttctgcgccaacc |
| PaDPEase_01 R | tgctcgagtgcggccgcTCAGACGACGTTGTGCGTCTTCATCC |
| PaDPEase_02 F | TGGACAGCAAATGGGTCGCatgccccgcttcgccgccaacc |
| PaDPEase_02 R | tgctcgagtgcggccgcTCATCCGTCGCGTCGAGTAGCGCG |
| BtDPEase F | TGGACAGCAAATGGGTCGCatgaaatattcgctatgtacc |
| BtDPEase R | tgctcgagtgcggccgcTTACGAAGTTACTACTTCTAATTTTC |
| GI_GP_Fusion F | ACAGCAAATGGGTCGCGGATCCATGTCTCTTACTACTGCTTCTTC |
| GI_GP_Fusion R | tagtagataccgtgtttaccacgagcacctaataagtgttcg |
| GI_GS1P_Fusion R | tagtagataccgtgtttacttccgcctccaccaccacgagcacctaataagtgttcg |
| GS2P R1 | ccacttccgcctccaccaccacgagcacctaataagtgttcg |
| GS2P F2 | GGTGGAGGCGGAAGTGGCGGTGGTGGCAGCAAACACGGTATCTACTACGCTTAC |
| DPEase_GP_Fusion R2 | tgctcgagtgcggccgcaagctttcattaagagtgtttgtgac |
| GS3P R1 | cacttccgcctccacctgaacctccccccccaccacgagcacctaataagtgttcg |
| GS3P F2 | AGGTGGAGGCGGAAGTGGCGGTGGTGGCAGCAAACACGGTATCTACTACGCTTAC |
| GI_GE1P_Fusion R | tagtagataccgtgtttCTTGGCAGCTGCTTCaccacgagcacctaataagtgttcg |
| GE2P R1 | tccttggcagctgcttcaccacgagcacctaataagtgttcg |
| GE2P F2 | GAAGCAGCTGCCAAGGAGGCAGCTGCGAAGAAACACGGTATCTACTACGCTTAC |
| GE3P R1 | ccttggcagctgcttctttcgcagctgcctcaccacgagcacctaataagtgttcg |
| GE3P F2 | AGAAGCAGCTGCCAAGGAGGCAGCTGCGAAGAAACACGGTATCTACTACGCTTAC |

**Table S2 Fusion proteins developed in this study**

| **Fusion protein** | **Linker** | **Amino acid sequence of linker** | **Encoding sequence of linker (5’ - 3’)** |
| --- | --- | --- | --- |
| GP^(1)^ | - | - | - |
| GS_1_P^(2)^ | (GGGGS)_1_ | GGGGS | GGTGGAGGCGGAAGT |
| GS_2_P | (GGGGS)_2_ | GGGGSGGGGS | GGTGGAGGCGGAAGTGGCGGTGGTGGCAGC |
| GS_3_P | (GGGGS)_3_ | GGGGSGGGGSGGGGS | GGGGGGGGAGGTTCAGGTGGAGGCGGAAGTGGCGGTGGTGGCAGC |
| GE_1_P^(3)^ | (EAAAK)_1_ | EAAAK | GAAGCAGCTGCCAAG |
| GE_2_P | (EAAAK)_2_ | EAAAKEAAAK | GAAGCAGCTGCCAAGGAGGCAGCTGCGAAG |
| GE_3_P | (EAAAK)_3_ | EAAAKEAAAKEAAAK | GAGGCAGCTGCGAAAGAAGCAGCTGCCAAGGAGGCAGCTGCGAAG |

(1) G refers to enzyme AcceGI; P refers to enzyme CcDPEase.

(2) S refers to the flexible linker GGGGS, while the subscript number refers to the repeating times. For instance, S_3_ refers to “GGGGSGGGGSGGGGS”, which containing three tandem GGGGS.

(3) E refers to rigid linker EAAAK, while the subscript number refers to the repeating times. For instance, E_3_ refers to EAAAKEAAAKEAAAK, which containing three tandem EAAAK.


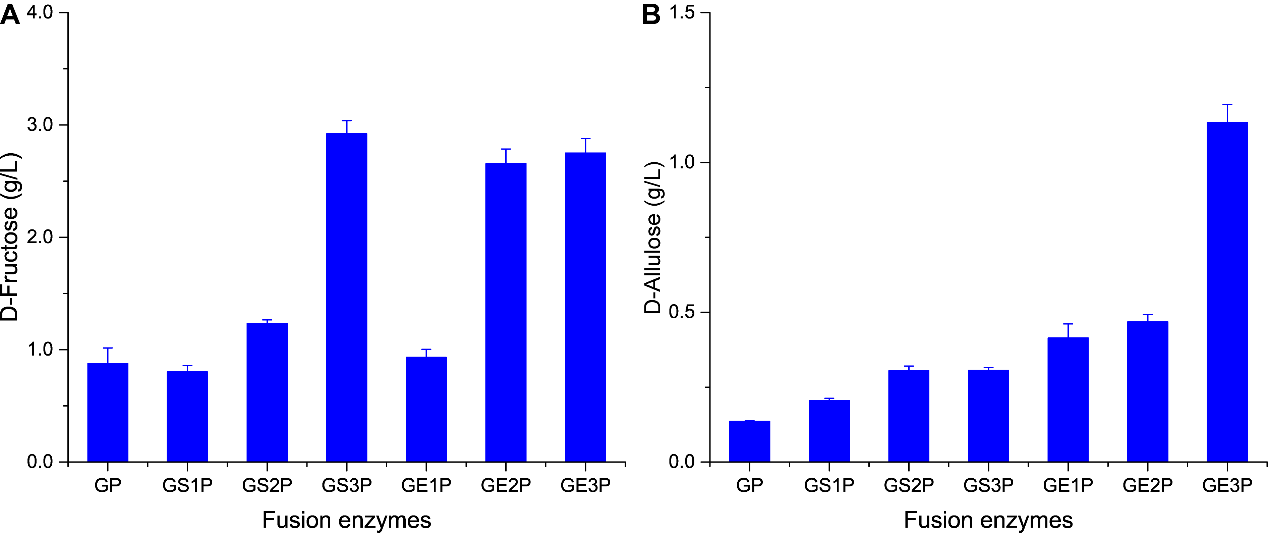


**Figure S1 Activity analysis of fusion enzymes.**

(A) Glucose isomerase activity analysis by converting D-glucose to D-fructose. (B) D-psicose 3-epimerase activity analysis by converting D-fructose to D-psicose. GP refers to the fusion enzyme linking AcceGI and CcDPEase directly. Other fusion proteins refer to those linking AcceGI and CcDPEase through corresponding flexible or rigid linkers.


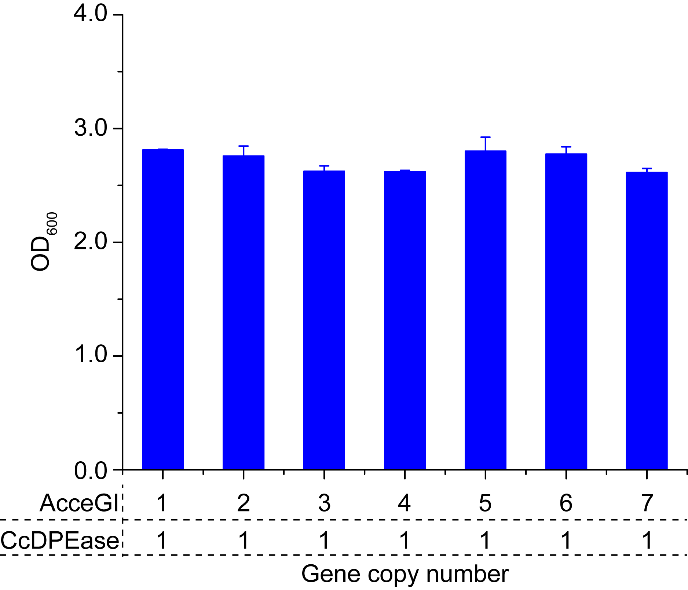


**Figure S2 Effect of overexpressing AcceGI on host cell biomass.**

The optical densities at 600 nm (OD_600_) of the host strains overexpressing 1 copy *CcDPEase* and 1–7 copies of *AcceGI* were measured 4 h after IPTG induction.
